# Pharmacokinetics, Bioavailability, Excretion and Metabolism Studies of Akebia Saponin D in Rats: Causes of the Ultra-Low Oral Bioavailability and Metabolic Pathway

**DOI:** 10.3389/fphar.2021.621003

**Published:** 2021-04-15

**Authors:** Pengfei Li, Jun Peng, Yuexin Li, Lili Gong, Yali Lv, He Liu, Tianhong Zhang, Song Yang, Hongchuan Liu, Jinglai Li, Lihong Liu

**Affiliations:** ^1^ Pharmacy Department of Beijing Chaoyang Hospital, Capital Medical University, Beijing, China; ^2^ Guollence Pharmaceutical Technology Co., Ltd., Beijing, China; ^3^ School of Pharmacy, Shenyang Pharmaceutical University, Benxi City, China

**Keywords:** Akebia saponin D, pharmacokinetics, bioavailability, metabolism, excretion, LC–MS/MS

## Abstract

**Background:** Akebia saponin D (ASD) has a variety of biological activities and great medicinal potential, but its oral bioavailability is so low as to limit its development. Its pharmacokinetic profiles and excretion and metabolism *in vivo* have not been fully elucidated. This study was an attempt in this area.

**Methods:** A simple LC-MS/MS method to simultaneously quantify ASD and its metabolites M1∼M5 in rat plasma, feces, urine and bile was established with a negative ESI model using dexketoprofen as the internal standard. Meanwhile, the UPLC-HR/MS system was used to screen all possible metabolites in the urine, feces and bile of rats, as compared with blank samples collected before administration. Absolute quantitative analysis was for M0, M3, M4, and M5, while semi-quantitative analysis was for M1, M2, and Orbitrap data.

**Results:** The AUC_0-t_ values after intravenous administration of 10 mg/kg and intragastrical administration of 100 mg/kg ASD were 19.05 ± 8.64 and 0.047 ± 0.030 h*μg/ml respectively. The oral bioavailability was determined to be extremely low (0.025%) in rats. The exposure of M4 and M5 in the oral group was higher than that of M0 in the terminal phase of the plasma concentration time profile, and ASD was stable in the liver microsome incubation system of rats, but metabolism was relatively rapid during anaerobic incubation of intestinal contents of rats, suggesting that the low bioavailability of ASD might have been attributed to the poor gastrointestinal permeability and extensive pre-absorption degradation rather than to the potent first pass metabolism. This assertion was further verified by a series of intervention studies, where improvement of lipid solubility and intestinal permeability as well as inhibition of intestinal flora increased the relative bioavailability to different extents without being changed by P-gp inhibition. After intravenous administration, the cumulative excretion rates of ASD in the urine and bile were 14.79 ± 1.87%, and 21.76 ± 17.61% respectively, but only 0.011% in feces, suggesting that the urine and bile were the main excretion pathways and that there was a large amount of biotransformation in the gastrointestinal tract. Fifteen possible metabolites were observed in the urine, feces and bile. The main metabolites were ASD deglycosylation, demethylation, dehydroxylation, decarbonylation, decarboxylation, hydroxylation, hydroxymethylation, hydroxyethylation and hydrolysis.

**Conclusion:** The pharmacokinetics, bioavailability, metabolism and excretion of ASD in rats were systematically evaluated for the first time in this study. It has been confirmed that the ultra-low oral bioavailability is due to poor gastrointestinal permeability, extensive pre-absorption degradation and biotransformation. ASD after *iv* administration is not only excreted by the urine and bile, but possibly undergoes complex metabolic elimination.

## Introduction

**FIGURE 1:**
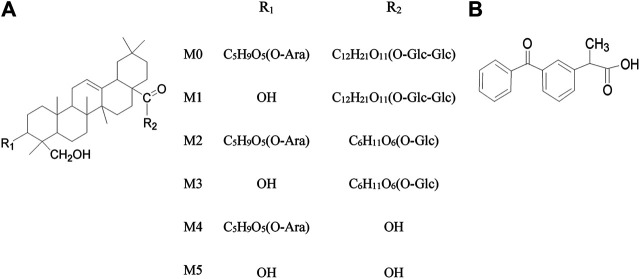
**(A)** Chemical structure of ASD and five metabolites **(B)** Dexketoprofen (IS).

Dipsacus asper Wall. ex C.B. Clarke, a well-known traditional Chinese herbal medicine, has long been used in China for treating bone diseases (e.g. bone fracture, osteoporosis, and rheumatic arthritis), low back pain, traumatic hematoma, uterine bleeding and those caused by the deficiency of the liver and kidney (Tao et al., 2020). The main chemical components include triterpenoid glycosides, iridoid glycosides, alkaloids and volatile oil compounds, which have been used in a variety of Chinese patent medicines (Chinese Pharmacopoeia Commission, 2005). Triterpenoid saponins possess a wide range of biological activities and are distributed in many important medicinal plants. Typically, Akebia saponin D (ASD, 3-O-α-l-arabinopyranosyl hederagenin-28-β-d-glucopyranoside (1→6-β-d-glucopyranoside), Figure 1A (M0)), also known as Asperosaponin VI, is a main bioactive triterpenoid saponin isolated from the rhizome of Dipsacus asper Wall (Song et al., 1993). In recent years, ASD has been reported to be a potential treatment drug for cancer (Jeong et al., 2008), Alzheimer's disease (Jeong et al., 2008; Zhou et al., 2009; Yu et al., 2012), cardiovascular disease (Li et al., 2010a; Li et al., 2010b; Li et al., 2012), and bone fractures (Peng et al., 2010; Niu et al., 2011). Moreover, our group has demonstrated that ASD can inhibit the deposition of lipids in the liver and the formation of the nonalcoholic fatty liver in ob/ob mice. ASD could also inhibit atherosclerosis by ameliorating metabolic disorders, and is regarded as a potential drug for hepatic steatosis (Gong et al., 2014; Gong et al., 2016; Gong et al., 2018; Yang et al., 2019).

Although ASD has a variety of biological activities and a great potential for medicine, the research on the pharmacological mechanism and the development of new drugs involving ASD has been somehow hampered by the incomplete metabolite spectrum *in vivo* and the ultra low absolute oral bioavailability (Wu et al., 2014; Liu et al., 2014). There have been few studies that have identified the full metabolites of ASD or accounted for the low bioavailability (Zhou et al., 2019; Yan et al., 2014). As a glucoside compound, ASD has been merely reported to undergo glycosidic bond hydrolysis into five main metabolites in rats, such as hederagenin-28-β-D-glucopyranoside-(1→6)-β-D-glucopyranoside, 3-O-α-L-Arabinopyranosyl hederagenin-28-β-D-glucopyranoside, hederagenin-28-β-D-glucopyranoside, 3-*O*-α-*L*-arabinopyranosyl hederagenin and hederagenin (Figure 1A (M1∼M5)) (Li et al., 2009). Oral gavage of ASD remodeled the composition of the gut microbiota, impacted the serum metabolomics, and had anti-hyperlipidemia effect that induced high fat diet in rats (Zhou et al., 2019). Given the extremely low exposure of the parent drug *in vivo* after oral administration, we speculated that 1) the pharmacological effects of ASD may be derived from parent drugs and metabolites, intermediate or final. 2) the gut microbiota might be not only the pharmacological target, but also the main site of metabolism, resulting in a low oral bioavailability.

To substantiate this speculation, we presented a simple LC–MS/MS method using the negative ESI mode for the simultaneous quantification of ASD and metabolites in rat plasma, feces, urine and bile with dexketoprofen as the internal standards (Figure 1B). The validated method was successfully applied to a systematic pharmacokinetic study in rats. Meanwhile, the UPLC-HR/MS system was used to screen all possible metabolites in the urine, feces and bile, and compared them with blank samples collected before administration. The design of the study included 1) an *in vitro* investigation of metabolic stability of ASD in rat liver microsomes and intestinal contents; 2) a comparative evaluation of pharmacokinetic behaviors and relative bioavailability of ASD *via* intragastrical administration to rats pretreated with vegetable oil (lipid solubility enhancement), metronidazole (intestinal flora inhibitors), verapamil (P-glycoprotein inhibitors) and urea (penetration enhancers); 3) an exploration of excretion kinetics in rat feces, urine and bile; 4) an identification of the metabolic pathways of ASD using UPLC-HR/MS method. The study was intended to gain insights into the pharmacokinetic mechanism of ASD so as to contribute to further research and clinical use of ASD.

## Materials and Methods

### Chemicals and Reagents

ASD (99.64% purity) and hederagenin-28-β-D-glucopyranoside (98.52% purity) were purchased from Chengdu Desite Biotech Co., Ltd (Chengdu, China). Cauloside A (97% purity) was purchased from Yunnan Xili Biotechnology Co., Ltd (Yunnan, China). Hederagenin (≥98% purity) was purchased from Shaihai Aladdin Biochemical Technology Co., Ltd (Shanghai, China). Dexketoprofen (≥98% purity) was purchased from National Institutes for Food and Drug Control (Beijing, China). Verapamil hydrochloride (100% purity) was purchased from SIGMA–ALDRICH (Shanghai, China). Verapamil hydrochloride Tablets were purchased from The Central Pharmaceutical Co., Ltd (Tianjin, China). Nicotinamide Adenine Dinucleotide Phosphate (98.5% purity) was purchased from Beijing Balinway Technology Co., Ltd (Beijing, China). Rat liver microsomes were purchased from Research Institute for Liver disease Co., Ltd (Shanghai, China). Metronidazole Tablets were purchased from Huazhong Pharmaceutical Co., Ltd (Xiangyang, China). Urea (analytical grade) was purchased from Sinopharm Group Chemical Reagent Co. Ltd (Shanghai, China). Methanol (HPLC grade) and Acetonitrile (HPLC grade) were purchased from Merck KGaA (Darmstadt, Germany). Formic acid (analytical grade) and ammonium formate were (LC grade) purchased from Thermo Fisher Scientific (Pittsburgh, United States). Vegetable oil was purchased from Beijing Wumart Commercial Group Co., Ltd (Beijing, China). All the other chemicals used in the preparation of samples were of reagent grade or better (Sigma) unless otherwise specified.

### Animals

All the animal experiments were conducted in line with the instructions of the Laboratory Animal Management Statute of China Physiological Society and were approved by the Beijing Chaoyang Hospital Committee on Ethics in the Care and Use of Laboratory Animals (IACUC#:2019004). Sprague–Dawley rats (220–260 g, male) were obtained from Beijing Vital River Laboratory Animal Technology Co., Ltd. (Beijing, China). Following arrival, the animals were kept in an environmentally controlled breeding room for one week before start of the experiments, with standard laboratory food and water available ad libitum. Food was withheld at 12 h before drug administration.

### Instrumentation

The LC–MS/MS system consisted of a Waters Acquity UPLC system (Waters Corp., Milford, MA, United States) and coupled with an AB SCIEX triple quadrupole mass spectrometer (Sciex Corp., Framingham, MA, United States). The ACQUITY UPLC platform coupled with a binary pump, a thermostated column compartment, an autosampler and an online degasser, was used for analyzing the ASD and metabolites. The MS/MS platform was coupled with Sciex Triple Quad 6,500 + mass spectrometer equipped with an electrospray ionization (ESI) source operated in a negative ion mode. Equipment control, data and analysis were acquired and processed using the Analyst 1.6 software supplied by Sciex.

The LC–HR/MS system consisted of a DIONEX Ultimate 3000 UHPLC system and coupled with a Q-Exactive mass spectrometer (Thermo Corp., Pittsburgh, PA, United States). The UPLC platform was composed of a binary pump solvent management system, an online degasser and an autosampler. The HR/MS platform was coupled with a Quadrupole Orbitrap mass spectrometer equipped with an electrospray ionization (ESI) source and HESI probe operated in both a positive and negative ion mode. The signal acquisition and peak integration were performed using Xcalibur 3.0 Software and Metworks 1.3 Software supplied by Thermo Fisher Scientific.

### Chromatographic Conditions

For quantitative analysis, chromatographic separations were performed on a Unitary C_8_ analytical column (50 mm × 2.1 mm i. d. 5 μm; ACCHROM, Beijing, China) and column temperature was maintained at room temperature for the ACQUITY UPLC platform. Solvent A (0.1% formic acid in water) and solvent B (acetonitrile) were used as mobile phases. The gradient elution program was optimized as follows: from 10 to 95% phase B at 0–2.00 min; 95% phase B maintained at 2.00–3.00 min; from 95 to 10% phase B at 3.00–3.01 min, followed by re-equilibration at 10% phase B until 4 min and the flow rate was 0.5 ml/min. Autosampler temperature was kept at room temperature and the injection volume was set at 4 μl for plasma, feces, urine samples and 6 μl for bile samples.

For qualitative analysis, chromatographic separations were performed on a Hypersil Gold C_18_ column (100 mm × 2.1 mm i. d. 3 μm; Thermo, PA, United States) and column temperature was maintained at room temperature for the RSLC platform. Solvent A (0.1% formic acid in water with 2 mm ammonium formate) and solvent B (acetonitrile) were selected as mobile phases. The gradient elution program was optimized as follows: 5% phase B at 0–1.00 min; from 5 to 95% phase B at 1–15.00 min; from 95 to 5% phase B at 15.00–20.00 min, followed by re-equilibration at 5% phase B until 25 min and the flow rate was 0.3 ml/min. Autosampler temperature was kept at room temperature and the injection volume was set at 5 μl.

### MS/MS Conditions

Analytes were detected by MS/MS with an electrospray ionization (ESI) interface in negative multiple reaction monitoring. The optimal settings of the MS parameters and m/z transitions were listed in Table 1. Full scan and Full MS/dd-MS2 mode were used for monitoring the ASD and all its metabolites by HR/MS and the instrument-dependent parameter settings for the mass spectrometer were listed in Table 2.

**TABLE 1:** Optimized mass spectrum parameters for quantification of ASD, internal standards and metabolites (M1–M5).

Analytes	Mw (Da)	Predecessor ion	Q1 (m/z)	Q3 (m/z)	DP (V)	CE (eV)	CXP (V)	EP (V)	CUR (psi)	GS1 (psi)	GS2 (psi)	ISV (V)	TEM (°C)
M0	929.10	[M+HCOO]^-^	973.6	603.3	−80	−50	−13	−10	20	45	45	−4,500	400
M3	634.84	[M+HCOO]^-^	679.4	471.3	−100	−25	−13	−10	20	45	45	−4,500	400
M4	604.81	[M+HCOO]^-^	649.4	603.4	−30	−35	−13	−10	20	45	45	−4,500	400
M5	472.70	[M-H]^-^	471.4	393.3	−160	−55	−25	−10	20	45	45	−4,500	400
M1	796.98	[M+HCOO]^-^	841.4	471.4	−100	−38	−13	−10	20	45	45	−4,500	400
M2	766.95	[M+HCOO]^-^	811.4	603.4	−100	−38	−13	−10	20	45	45	−4,500	400
IS	254.28	[M-H]^-^	253.2	209.1	−10	−10	−18	−10	20	45	45	−4,500	400

Q1: precursor ion; Q3: product ion; DP: declustering potential; EP: entrance potential; CE: collision energy; CXP: collision exit potential; CUR: curtain gas; GS1: nebulizer gas; GS2: heater gas; ISV: ion spray voltage; TEM: ion spray temperature; IS: internal standards.

**TABLE 2:** Instrument-dependent parameters settings for qualification of ASD and metabolites.

	Sheath gas (Arb)	Aux gas (Arb)	ISV (V)	P-TEM (°C)	C-TEM (°C)	RE	Scan rang (m/z)	AGC target	Maximum TT (ms)	NCE
Full scan	35	10	3,000	350	350	70,000	70–1,050	1e6	100	/
Full MS/dd-MS2	35	10	3,000	350	350	35,000	/	1e5	50	20,40,60

Aux gas: Auxiliary gas; ISV: spray voltage; P-TEM: probe heater temperature; C-TEM: capillary temperature; RE: Resolution.

### Preparation of Calibrators and Quality Control Samples

ASD (M0), hederagenin-28-β-d-glucopyranoside (M3), cauloside A (M4) and hederagenin (M5) were dissolved in methanol to produce 1 mg/ml calibration standard (CS) and quality control (QC) stock solution respectively, and stored at 4°C. The combined working solutions of analytes in the desired concentration range were prepared by appropriate dilution of CS and QC stock solutions with methanol/water (1/1, v/v). The above working solutions were spiked to the drug-free biological matrix at a maximum allowable limit of 5% for blank plasma, urine, fecal homogenates and 10% for the bile to obtain the respective CS or QC samples. The linear ranges of M0, M3, M4, and M5 in plasma were 0.1–500, 0.2–1,000, 0.2–1,000, and 1–5,000 ng/ml, respectively, compared to 1–500 ng/ml in the urine, 20–10,000 ng/g in fecal homogenates and 5–2000 ng/ml in the bile, respectively. Stock solutions of dexketoprofen (IS) were prepared independently at a concentration of 1 mg/ml. The solutions were diluted with methanol/water(1/1, v/v) to achieve concentrations of 1 μg/ml of dexketoprofen for plasma analysis and 2 μg/ml of dexketoprofen for fecal homogenates, urine and bile analysis.

### Sample Preparation

Blank matrices of rat plasma, urine, feces and bile were obtained from drug free healthy rats. Under carbon dioxide anesthesia, blood samples were withdrawn from the orbital sinus into K2EDTA-treated tubes and centrifuged at 2000 g for 15 min at 4°C to obtain the plasma fractions and stored at −20°C. After collection, the fecal specimens were dried and weighed, homogenized by addition of twenty-fold volume of methanol/water (1/4, v/v), and sonicated for 15 min. The fecal homogenate was then centrifuged at 2000 g for 10 min at 4°C, and the supernatant was stored at −70°C. The bile and urine were stored at −70°C until analyses.

### Plasma Procedure

All the plasma samples were allowed to thaw at room temperature and homogenized *via* vortex. The rat plasma pretreatment for analytes involved simple protein precipitation with methanol. An aliquot of 50 μl IS working solution (dexketoprofen, 1 μg/ml) was added to the plasma sample of 100 μl. A volume of 350 μl methanol was added to the mixture and vortexed for 1 min to enhance precipitation and extracted by centrifugation at 20,200 g for 10 min at 4°C. 100 μl of the supernatant without filtration was transferred to the auto-sampler vial and 4 μl was injected into the LC–MS/MS system. For the double blank samples, 95 μl of blank plasma was added to 405 μl of methanol, which was mixed *via* vortex for 1 min and centrifuged at 20,200 g for 10 min at 4°C. For the QC_0_ samples, 95 μl of blank plasma was added to 355 μl of methanol and 50 μl of IS (dexketoprofen, 1 μg/ml), which was mixed *via* vortex for 1 min and centrifuged at 20,200 g for 10 min at 4°C. For the calibration standard and quality control samples, 5 μl aliquot of each CS and QC solutions was combined with 95 μl of blank plasma, vortexed for 30 s before 50 μl of IS working solution (dexketoprofen, 1 μg/ml) and 350 μl of methanol were added to the plasma samples, which were mixed via vortex for 1 min and centrifuged at 20,200 *g* for 10 min at 4°C. 4 μl of aliquot of the supernatant was injected into the LC–MS/MS system for analysis.

### Urine and Feces Procedure

All the fecal homogenate and urine samples were allowed to thaw at room temperature and homogenized *via* vortex. The fecal homogenate and urine pretreatment for analytes involved simple protein precipitation with methanol. An aliquot of 50 μl IS working solution (dexketoprofen, 2 μg/ml) was added to 100 μl of fecal homogenate or urine samples, respectively. A volume of 850 μl methanol was added to the mixture and vortexed for 1 min to enhance precipitation and extracted by centrifugation at 20,200 g for 10 min at 4°C. The supernatant was transferred to the auto-sampler vial and 4 μl was injected into the LC–MS/MS system. For the double blank samples, 95 μl of blank fecal homogenate or urine was added to 905 μl of methanol, which was mixed *via* vortex for 1 min and centrifuged at 20,200 *g* for 10 min at 4°C, respectively. For the QC_0_ samples, 95 μl of blank fecal homogenate or urine was added to 855 μl of methanol and 50 μl of IS (dexketoprofen, 2 μg/ml), which was mixed *via* vortex for 1 min and centrifuged at 20,200 *g* for 10 min at 4°C, respectively. For the calibration standard and quality control samples, 5 μl aliquot of each CS and QC solutions was combined with 95 μl of fecal homogenate or urine, respectively. The samples were vortexed for 30 s before 50 μl of IS working solution (dexketoprofen, 2 μg/ml) and 850 μl of methanol were added to the samples, which were mixed *via* vortex for 1 min and centrifuged at 20,200 *g* for 10 minat 4°C. 4 μl aliquot of the supernatant was injected into the LC–MS/MS system for analysis.

### Bile Procedure

All the bile samples were allowed to thaw at room temperature and homogenized *via* vortex. The bile pretreatment for analytes involved simple protein precipitation with methanol. An aliquot of 50 μl IS working solution (dexketoprofen, 2 μg/ml) was added to the bile sample of 50 μl. A volume of 900 μl methanol was added to the mixture and vortexed for 1 min to enhance precipitation and extracted by centrifugation at 20,200 g for 10 min at 4°C. The supernatant was transferred to the auto-sampler vial and 6 μl was injected into the LC–MS/MS system. For the double blank samples, 45 μl of blank bile was added to 955 μl of methanol, which was mixed *via* vortex for 1 min and centrifuged at 20,200 g for 10 min at 4°C. For the QC_0_ samples, 45 μl of blank bile was added to 905 μl of methanol and 50 μl of IS (dexketoprofen, 1 μg/ml), which was mixed *via* vortex for 1 min and centrifuged at 20,200 g for 10 min at 4°C. For the calibration standard and quality control samples, 5 μl aliquot of each CS and QC solutions was combined with 45 μl of blank bile. The bile samples were vortexed for 30 s before 50 μl of IS working solution (dexketoprofen, 1 μg/ml) and 900 μl of methanol were added to the bile samples, which were mixed *via* vortex for 1 min and centrifuged at 20,200 g for 10 min at 4°C. 6 μl aliquot of the supernatant was injected into the LC-MS/MS system for analysis.

## Method Validation

Validation of the presented method regarding specificity, linearity and lower limit of quantification was performed in rat plasma, fecal homogenates, urine and bile, according to the FDA guidelines for validation of bioanalytical methods (US Food and Drug Administration, Center for Drug Evaluation and Research, 2001).

**FIGURE 2:**
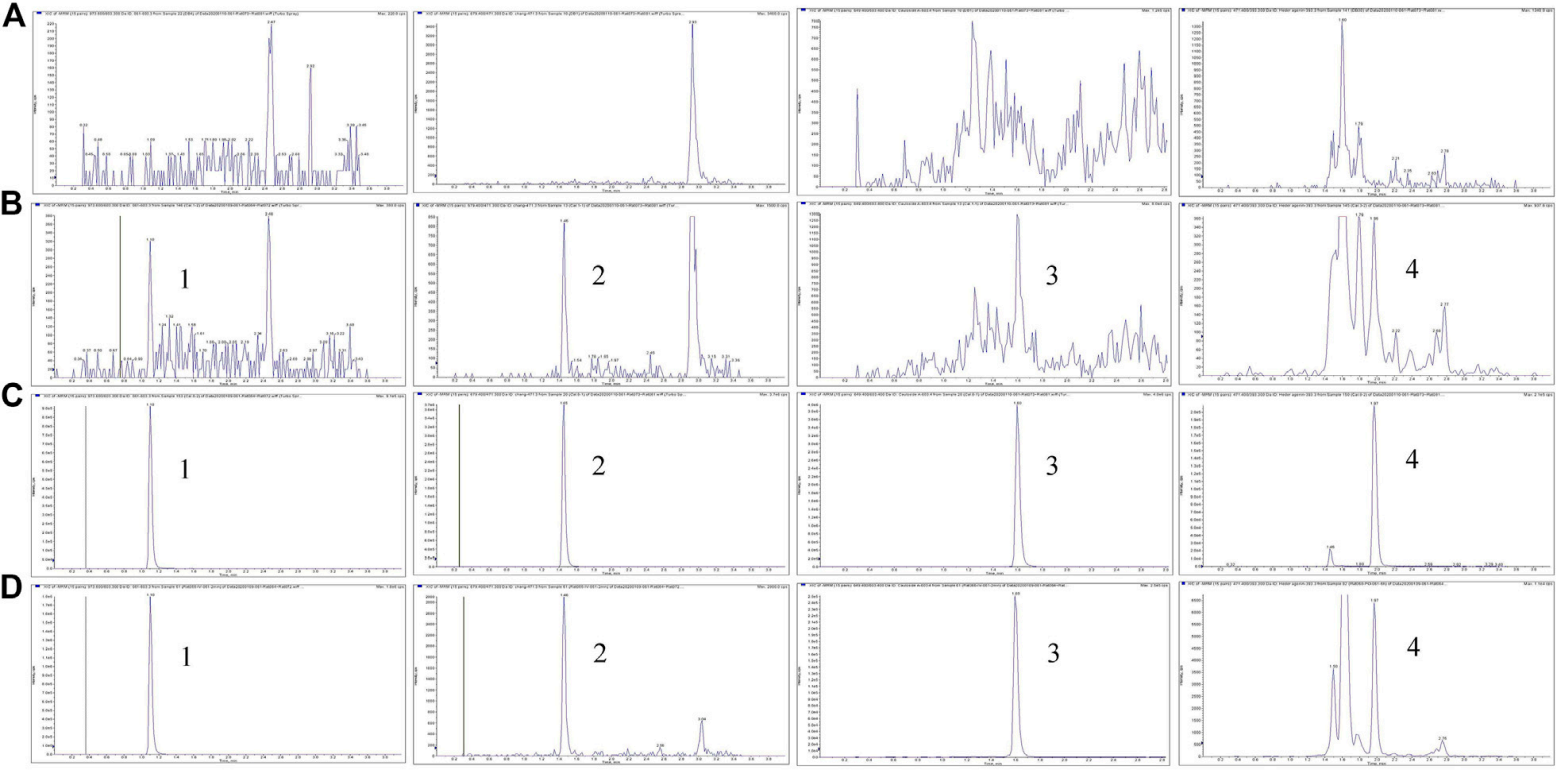
Representative MRM chromatograms for M0, M3, M4, and M5 **(A)** Blank plasma samples **(B)** LLOQ samples **(C)** ULOQ samples **(D)** Plasma samples after intravenous administration of ASD (10 mg/kg); Peak 1, M0 (t_R_ = 1.0 min); Peak 2, M3 (t_R_ = 1.46 min); Peak 3, M4 (t_R_ = 1.60 min); Peak 4, M5 (tR = 1.97 min).

The specificity was assessed by analyzing and comparing the chromatograms of blank plasma, fecal homogenates, the urine and bile with the corresponding spiked samples and study samples after dosing. Under LC-MS/MS conditions, there was no interfering peak during the elution of the analytes or IS in the blank matrices. Examples of the typical MRM chromatograms of the analytes in plasma are shown in Figure 2, which demonstrates that ASD (M0), M3, M4, and M5 were well separated and the peak shapes were satisfactory. The retention times of ASD (M0), M3, M4, and M5 were 1.10, 1.46, 1.60, and 1.97 min, respectively.

For quantification of ASD (M0), M3, M4, and M5 in plasma, urine, bile and fecal homogenate samples, standard curves of relevant concentrations for each standard substance were drawn. To evaluate the linearity, the calibration curves of all the analytes were established by an internal standard method using the *1/x* ^2^ weighted linear least-squares regression model. The curves displayed a good linearity characterized by regression coefficients of *r* = 0.99 or higher with a wide linear range, 5,000 times in plasma, 500 times in the urine, fecal homogenates and bile. This method is sensitive enough for quantitative detection of ASD and its metabolites.

### Metabolic Stability *in vitro*

*In vitro* metabolic study in liver microsomes is the most common approach to early estimation and prediction of *in vivo* metabolism. As was specified by the reported procedures (Amer et al., 2017; Meyer et al., 2012; Attwa et al., 2018), 100 µl of 2.5 μm ASD and 100 µlof 0.5 mg/ml RLMs, prepared with 10 mm phosphate buffer (pH 7.4), were mixed in a 1.1 ml microcentrifuge tube. The mixture was pre-warmed at 37°C in a water bath for 5 min, so was the freshly prepared NADPH. Then, 50 µl of the warmed NADPH was added into the mixture to initiate the metabolic reactions that were terminated using protein precipitation by drawing 20 µl of the incubation mixture into tubes containing 200 µl of ice-cold acetonitrile/methanol (1/1, v/v) at 0, 5, 15, 30, 60, 90, and 120 min followed by centrifugation at 3500 rpm for 10 min at 4°C. The supernatant was transferred to UPLC auto-sampler vials and analyzed according to the analytical method. This study was performed in triplicate for each time point and paralleled with verapamil as positive control.

The anaerobic incubation method of ASD with rat enterobacteria was employed as specified by literatures (Takagaki and Nanjo, 2010; Ke et al., 2016; Yan et al., 2018). The concentration of ASD, prepared with 10 mm phosphate buffer (pH 7.4), was 0.5 μm. An aliquot of 10 ml of 0.5 μm ASD was pre-warmed at 37°C in a water bath for 5 min before fresh intestinal contents of SD rats were added into the tube to initiate the reaction. The incubation mixture was divided into three Petri dishes. After incubation of 0, 0.5, 1, 2, 3, and 4 h, aliquots (0.2 ml) of the incubation mixture were withdrawn before 0.4 ml of ice-cold acetonitrile/methanol (1/1, v/v) containing 1 μg/ml of dexketoprofen was added to the reaction mixture. The experiment was carried out in an anaerobic glovebox under N_2_ atmosphere. The sample was centrifuged at 20200 g for 10 min at 4°C, transferred to UPLC auto-sampler vials and analyzed according to the analytical method.

### Dosing and Pharmacokinetic Study

To explore the mechanism of low bioavailability of ASD, ASD was intragastrically administrated with vegetable oil to improve solubility, pretreated metronidazole to inhibit intestinal flora, verapamil to inhibit P-glycoprotein (P-gp) and with urea to enhance intestinal penetration (Beig et al., 2016; Soto et al., 2018; Iwamoto et al., 2019; Liu et al., 2019). Thirty male rats were randomly divided into six groups (*n* = 5 per group) and fasted for 12 h before test drug administration. In Group A, ASD, dissolved in a saline solution (0.9% NaCl), was administered intravenously at a single dose of 10 mg/kg. In the other groups, ASD was administered intragastrically at a single dose of 100 mg/kg in a saline solution (0.9% NaCl) (Group B), in a saline solution (0.9% NaCl) of 50% vegetable oil (Groups C), to rats pretreated with metronidazole (2500 mg/kg, i. g., 3 times/day, 7 times in total) (Group D), or in a solution of verapamil (25 mg/kg, i. g., 3 times/day, 5 times in total) (Group E), and in a saturated solution of urea (25 mg/kg, i. g., one time in total) (Groups F). Serial blood samples were withdrawn from the rat jugular vein into K_2_EDTA-treated tubes under carbon dioxide anesthesia at 0.033 (only in Groups A), 0.083, 0.25, 0.5, 1, 2, 4, 6, 8, and 12 h after dosing. The plasma fractions were then separated by centrifugation at 2000 *g* for 10 min at 4°C, and stored at −20°C until analysis.

### Dosing and Excretion Study

To study fecal, urinary and biliary excretion, fifteen male rats were randomly divided into three groups (*n* = 5 per group) and fasted for 12 h before administration. ASD, dissolved in a saline solution (0.9% NaCl), was administered at a single dose of 10 mg/kg intravenously (Group A) and 100 mg/kg intragastrically (Group B) for fecal and urinary detection. The rats in Group C had their bile ducts cannulated under anesthesia by pentobarbital sodium (50 mg/kg) before being administered at a single dose of 10 mg/kg intravenously to detect biliary excretion. Urine and bile samples were collected at pre-dose, 0–6, 6–12, 12–24, and 24–48 h after dosing, while fecal samples were collected at pre-dose, 0–12, 12–24, and 24–48 h after dosing, which were homogenized with 20% methanol water solution at a ratio of 1:20 (w/v) after being weighed and dried. The precipitate was removed by centrifugation at 4°C, 20, 200 g for 10 min. All the biological samples were frozen at −70°C until assayed.

### Metabolic Transformation Study *in vivo*

In this study, a simple and sensitive qualitative method based on liquid chromatography combined with Q-Exactive-Orbitrap tandem mass spectrometry in both positive and negative ion modes (detailed parameters of detection can be seen in above methods) was established for the determination of ASD and metabolites in rat samples obtained in excretion study, including the fecal homogenate, urine and bile.

### Statistical Analysis

All data are expressed as mean ± SD. Noncompartmental analysis was performed using WinNonlin Phoenix version 8.0 (Cetera, Princeton, NJ) to calculate the pharmacokinetic parameters. Statistical analysis was performed using SPSS 18.0 (SPSS Inc., Chicago, IL, United States). After the outliers screening and exclusion, examination for the normal distribution was performed by the Kolomogorov–Smirnov test. One-way ANOVA followed by Tukey’s test was employed to compare the means of pharmacokinetic parameters between groups. *p* < 0.05 was considered statistically significant.

## Results and Discussion

### Metabolic Stability *in vitro*

**FIGURE 3:**
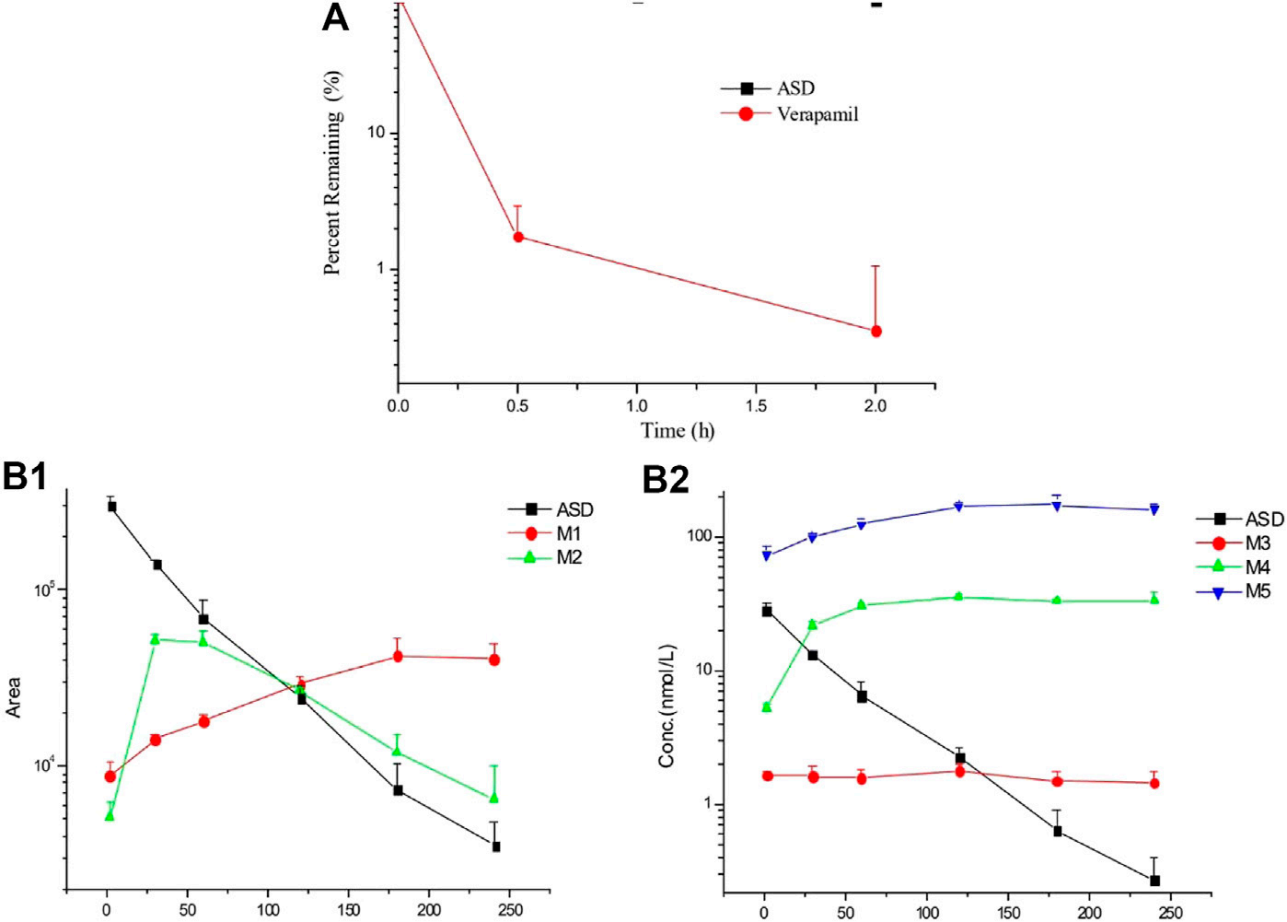
Elimination profiles of ASD and verapamil **(A)** in rat liver microsomes and metabolites **(B)** in rat intestinal flora (*n* = 3).

It is known that liver metabolism, especially CYP450 enzymes, contributes much to drug clearance and is considered one of the crucial factors for the systematic drug exposure, which was why the metabolic stability of ASD in rat liver microsomes was the first to be evaluated. The data are presented in a semi-log scale chart (Figure 3A) and each point represents the mean ± SD of three parallel samples. The results showed that the concentration of ASD had hardly changed during 2 h of incubation, but the metabolic conversion rate of the positive control drug verapamil exceeded 90% in the same period, suggesting that metabolism by liver related enzymes might not have been the main pathway for elimination of ASD, and that factors other than hepatic-intestinal first pass metabolism might have been involved in the low bioavailability of ASD.

After 4 h of incubation in the supernatant of intestinal contents of rats, a visible decrease was observed for ASD (M0), while metabolites (M3, M4, and M5) could be detected **(** Figure 3B **)**. It is worth mentioning that anaerobic conditions were critical to this experiment. If the experiment had been performed under natural conditions, ASD would have been stable and no metabolite could have been detected. Collectively, biotransformation of ASD might have been influenced by regulation of the composition and activities of intestinal microbiota. According to the results of this study, the ultra-low oral bioavailability of ASD might have been due to biotransformation in intestinal flora.

### Pharmacokinetic Study

**FIGURE 4:**
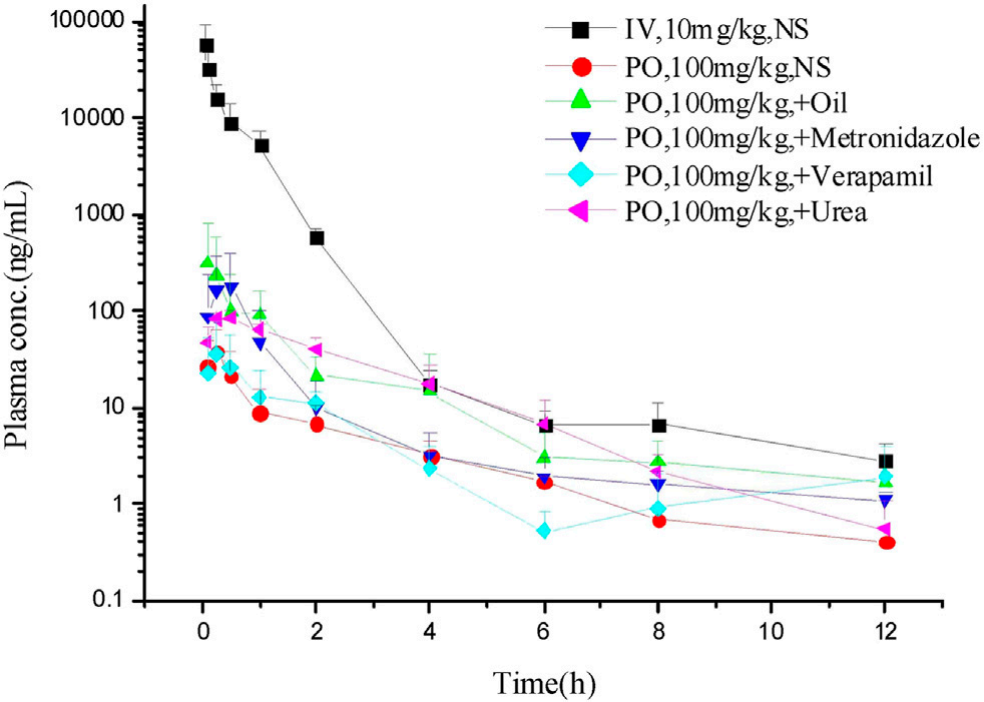
Mean plasma concentration-time profiles of ASD in different groups (*n* = 5).

**FIGURE 5:**
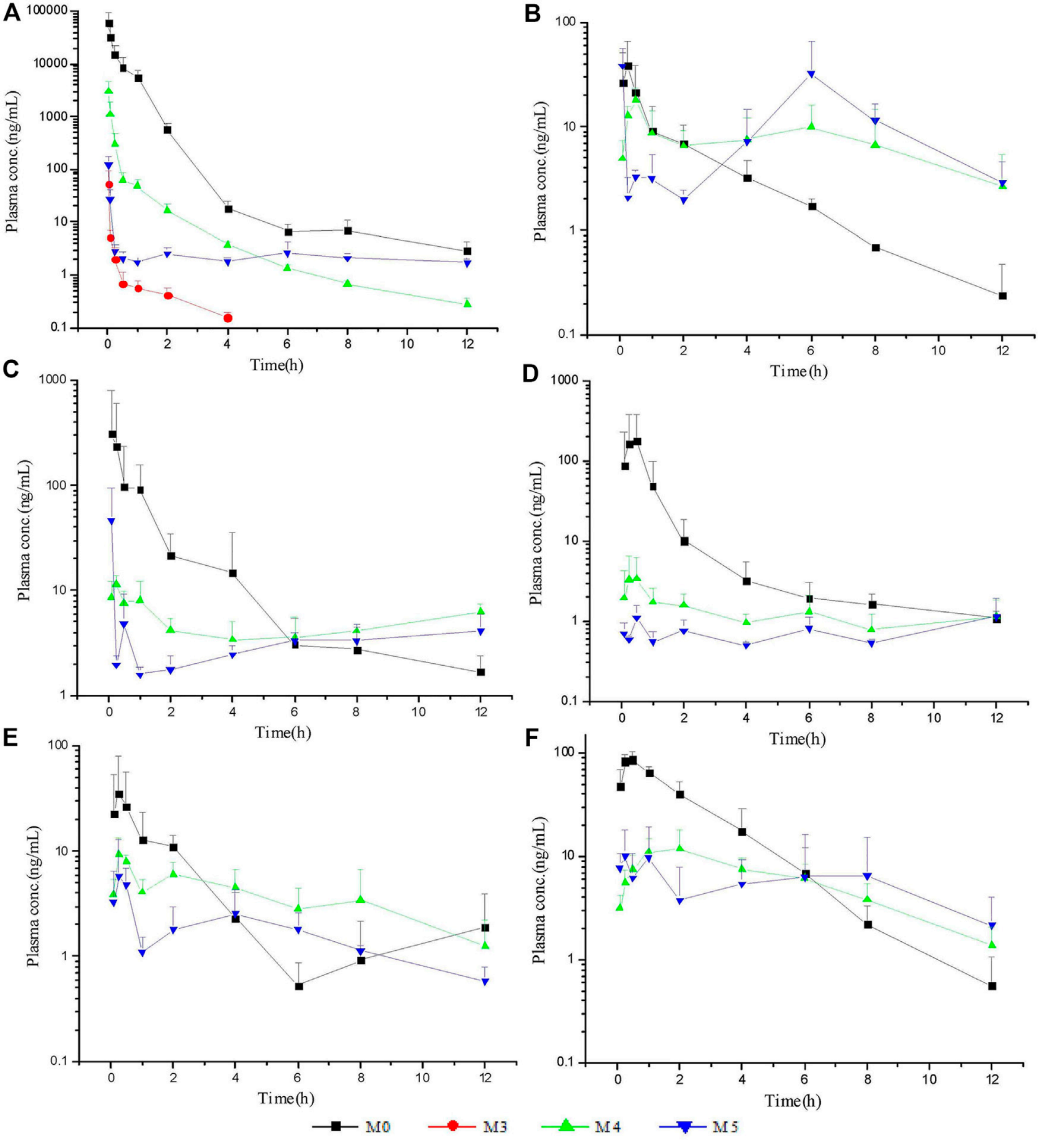
Mean plasma concentration-time profiles of ASD and metabolites: intravenous administration **(A)** at a dose of 10 mg/kg; intragastrical administration **(B)** and combined intragastrical administration with vegetable oil **(C)**, metronidazole **(D)**, verapamil **(E)**, urea **(F)** at a dose of 100 mg/kg (*n* = 5).

The LC–MS/MS methods which had been developed and validated were successfully applied to the pharmacokinetic study, and the quantitative range proved to be appropriate. The mean plasma concentration-time profiles of ASD (M0), M3, M4 and M5 in different groups are presented in Figures 4, 5, and the corresponding pharmacokinetic parameters calculated by non-compartmental analysis are summarized in Table 3.

**TABLE 3:** Pharmacokinetic parameters and bioavailability of ASD in rats after intravenous and intragastrical administration (*n* = 5).

Parameter	Administration	Parameter value (Mean ± SD)					
		IV, 10 mg/kg NS	PO, 100 mg/kg NS	PO, 100 mg/kg + Vegetable oil	PO, 100 mg/kg + Metronidazole	PO, 100 mg/kg + Verapamil	PO, 100 mg/kg + Urea
t_1/2_	(h)	2.54 ± 1.60	2.27 ± 0.60	3.01 ± 2.04	4.79 ± 0.79	2.00 ± 0.85	1.58 ± 0.23
T_max_	(h)	0.03 ± 0.00	0.25 ± 0.00	0.44 ± 0.49	0.33 ± 0.14	0.83 ± 1.01	0.42 ± 0.14
C_max_	(μg/ml)	59.40 ± 33.54	0.039 ± 0.027	0.34 ± 0.48	0.18 ± 0.21	0.036 ± 0.044	0.092 ± 0.017*
Vz	(L/kg)	2.35 ± 1.90	8,099 ± 4,870	3,214 ± 2,612	11,061 ± 13,466	6,220 ± 4,274	1,037 ± 133
Cl	(L/h/kg)	0.63 ± 0.37	2,376 ± 1,014	709 ± 547	1,428 ± 1,594	2,376 ± 1,381	461 ± 91
MRT_(0-t)_	(h)	0.50 ± 0.06	1.90 ± 0.11	1.97 ± 0.61	2.33 ± 1.86	2.14 ± 0.27	2.14 ± 0.40
AUC_(0-t)_	(h*μg/ml)	19.05 ± 8.64	0.047 ± 0.030	0.27 ± 0.30	0.18 ± 0.20	0.058 ± 0.047	0.22 ± 0.043*
AUC_(0-∞)_	(h*μg/ml)	19.06 ± 8.65	0.050 ± 0.028	0.28 ± 0.30	0.19 ± 0.20	0.063 ± 0.054	0.22 ± 0.044*
F	%	**/**	0.025	0.14	0.094	0.030	0.12

NC: Not calculated; t_1/2_: Terminal half-life; C_max_: Peak plasma concentration; T_max_: Time of C_max_; Vz: The apparent volume of distribution; Cl: Clearance; MRT: Mean Residue Time; AUC: Area under the curve.*p < 0.05 vs. PO 100 mg/kg NS group.

After intravenous administration of 10 mg/kg ASD, metabolites M3, M4, and M5 were detected in rat plasma with the systemic exposure lower than M0. Although M1, and M2 were not quantitatively recorded for lack of reference standards, they were also monitored in plasma and exhibited MS response comparable to M3. ASD might have undergone a complex sequential hydrolysis process *in vivo* , which might have contributed partly to systemic clearance.

The AUC_0-t_ value of intragastrical administration of 100 mg/kg ASD was 0.047 ± 0.030 h*μg/ml. As compared to data obtained from i. v. group, the oral bioavailability was determined to be extremely low (0.025%) in rats, based on the assumption of linear pharmacokinetics. Considering that ASD is stable to P450 enzymes, characterized by high solubility, low permeability and classified as BCS 3 compound, the low bioavailability could be attributed to the poor gastrointestinal absorption rather than to potent first pass metabolism. In the Caco-2 cell monolayer model, the Papp values of many other natural products, such as paeoniflorin, liquiritin, dammarane saponins and ginsenoside, were less than 1 × 10^–6^ cm/s according to literature. These products have poor bioavailability due to their low permeability (Liu et al., 2008; Zhang et al., 2010; Zhao et al., 2010; Ahmed et al., 2020). It is speculated that poor bioavailability of ASD arises from low permeability.

In rat plasma following intragastrical administration of 100 mg/kg ASD, M4, and M5 were detected with a comparable AUC level of M0, while M1, M2, and M3 concentrations were trace (data not shown). The higher exposure of M4, and M5 than M0 in the terminal phase of the plasma concentration time profile suggested that intense metabolic transformation might have occurred in the intestinal lumen, where the microbial and intestinal fluid environment facilitated M0 hydrolysis. The results of research on intestinal flora metabolism *in vitro* indicate that like many other natural products, gut microbiota might be not only the pharmacological target, but also the main metabolism site for ASD. According to literature, many other natural products like glycoside components need to be metabolized and deglycosylated by the intestinal flora to exert better pharmacological activity (Yan et al., 2018).

To further elucidate the mechanism of poor bioavailability and seek solutions, combined administrations with vegetable oil, urea, metronidazole, and verapamil were attempted to improve lipid solubility, intestinal permeability and inhibit intestinal microbiota and P-gp. As is shown in Table 3, the absorption of ASD was slightly improved in each combined dosing group. However, due to the huge variability, the C_max_ and AUC showed no statistically significant differences between groups except urea combined dosing group vs. NS group, suggesting that ASD might not have been a substrate of P-gp, and that the intestinal environment might have played an important role in absorption.

### Excretion Study

**FIGURE 6:**
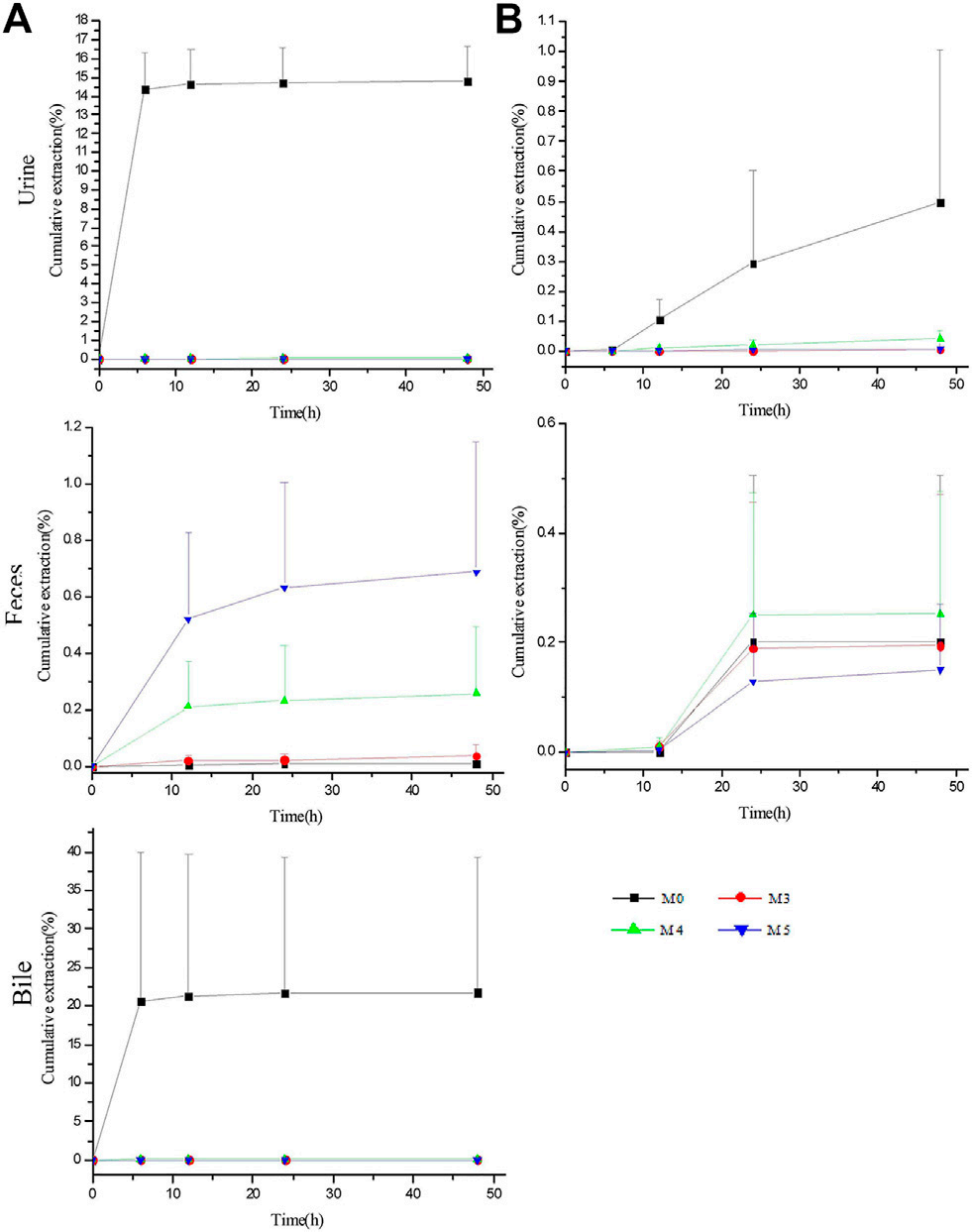
Biliary, urinary and fecal cumulative excretion profiles of ASD and its metabolites after intravenous **(A)** and intragastrical **(B)** administration (*n* = 5).

The excretion study of ASD and its metabolites was conducted following intravenous administration at a single dose of 10 mg/kg and intragastrical administration at a single dose of 100 mg/kg. The cumulative excretion amounts of M0, M3, M4, and M5 in the urine, feces and bile are shown in Figure 6.

For intravenous administration, the cumulative excretion amounts of M0 in the urine and bile rose rapidly within 6 h, reached the plateau gradually at 12 h and amounted to 14.79 ± 1.87% and 21.76 ± 17.61% within 48 h, respectively, suggesting that excretion by the urine and bile contributed greatly to the elimination of ASD. Unexpectedly, despite the large amounts of ASD drained into the GI tract, only 0.011% of the parent drug was excreted in the feces within 48 h. Meanwhile, the cumulative excretion amounts of M3, M4, and M5 were less than 0.014% in the urine and 0.1% in the bile, compared with 0.039 ± 0.041%, 0.26 ± 0.24%, and 0.69 ± 0.46% for M3, M4, and M5 in the feces, respectively.

For intragastrical administration, 0.26 ± 0.40% of M0 was excreted by the feces, marginally more than that of iv, and extremely lower urinary excretion than that of iv, suggesting that little ASD was absorbed into systemic circulation. Therefore, there is reason to postulate that ASD underwent an intensive biotransformation in GI lumen, whether administered intravenously or intragastrically.

However, the total recovery rate was lower than 40% in iv group and less than 1% in intragastrical group, suggesting the involvement of a large number of other metabolites *in vivo* that have not been identified or reported in literature.

### *In Vivo* Metabolism of ASD in Rats

The qualitative analysis of rat biological samples after intravenous administration of ASD was performed *via* LC-HR/MS system. Due to the high sensitivity and resolution UHPLC-Q-Exactive-Orbitrap-MS, a total of 15 compounds, including five metabolites reported in literature, were characterized in the urine, feces and bile of rats under positive and negative ion scanning patterns. The composition, accurate molecular weight, retention time and MS/MS fragments of these compounds were examined in detail *via* the combination of Metworks Version 1.3 software and putative annotation. The error results indicated that the reliability and precision for all the compounds and their product ion were less than ±5 ppm. The components detected in the biological samples of the rats and their relative abundance in the urine, feces and bile are listed in Table 4.

**TABLE 4:** Characterized metabolites in rat feces, urine and bile following intravenous injection of ASD at dosage of 10 mg/kg by UHPLC-Q-Exactive-Orbitrap-MS.

Peak ID	Putative metabolite	RT (min)	Mass shift	Theo. m/z	Meas. m/z	Error (ppm)	Molecular weight	Molecular formula	Biological source	Intensities of metabolites		
										Feces	Urine	Bile
M0	M	7.40	0.0000000	927.4958885	927.4929810	−3.1	929.10	C_47_H_76_O_18_	F,U,B	−	+++	+++
M1	[M-C_5_H_8_O_4_]	9.25	−132.0500811	795.4458074	795.4453061	−0.6	796.98	C_42_H_68_O_14_	F,U,B	++	++	+
M2	[M-C_5_H_8_O_5_]	9.40	−162.0528233	765.4430652	765.4422013	−1.1	766.95	C_41_H_66_O_13_	F,U,B	+	++	+
M3	[M-C_5_H_8_O_6_]	9.98	−294.0950819	633.4008066	633.4011802	0.6	634.84	C_36_H_58_O_9_	F,U,B	+	+	++
M4	[M-C_5_H_8_O_7_]	10.20	−324.1056466	603.3902419	603.3890381	−2.0	604.81	C_35_H_56_O_8_	F,U,B	++	++	++
M5	[M-C_5_H_8_O_8_]	11.80	−456.1479052	471.3479832	471.3478088	−0.4	472.70	C_30_H_48_O_4_	F,U,B	++	+	+
M6	[M+O]	6.59	15.9949146	943.4908031	943.4893188	−1.6	945.09	C_47_H_76_O_19_	F	++	ND	ND
M7	[M-CH_2_O]	10.35	−30.0105647	897.4853238	897.4847412	−0.6	899.07	C_46_H_74_O_17_	F	+	ND	ND
M8	[M-CH_2_]	6.67	−14.0156,501	913.4802384	913.4799805	−0.3	915.07	C_46_H_74_O_18_	F	+	ND	ND
M9	[M+CH_2_O]	8.88	30.0105647	957.5064531	957.5051270	−1.4	959.12	C_48_H_78_O_19_	F,U,B	+++	+	++
M10	[M+H_2_O]	17.11	18.0105647	945.5064531	945.5053711	−1.1	947.11	C_47_H_78_O_19_	F,U	++	+	−
M11	[M+C_2_H_5_O]	17.12	45.0340397	972.5299282	972.5313110	1.4	973.15	C_49_H_80_O_19_	F,U,B	+	+	−
M12	[M-CO]	15.61	−27.9949146	899.5009739	899.5025024	1.7	901.09	C_46_H_76_O_17_	F	+	ND	ND
M13	[M-O]	9.19	−15.9949146	911.5009739	911.4999390	−1.1	913.10	C_47_H_76_O_17_	F,U	++	++	ND
M14	[M+CH_2_]	9.01	14.0156501	941.5115385	941.5095215	−2.1	943.12	C_48_H_78_O_18_	F,U,B	−	++	++
M15	[M-CO_2_]	16.91	−43.9898292	883.5060593	883.5031738	−3.3	885.09	C_46_H_76_O_16_	B	ND	ND	−

ND: Not detected. It is based on the intensities of typical ions in the mass spectra and ranges from high (+++) to trace (-).

**FIGURE 7:**
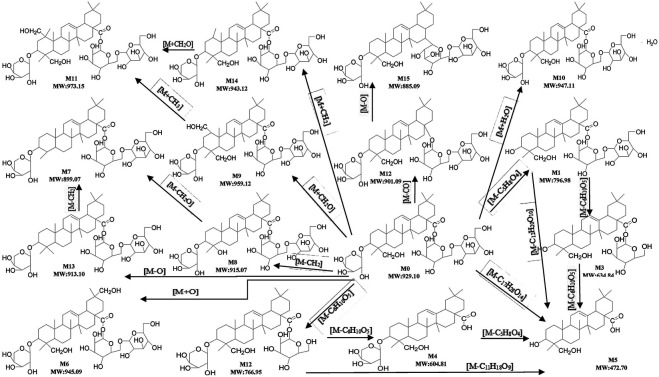
Proposed metabolic pathways of ASD in rat urine, feces and bile.

In this study, all plausible metabolites in the urine, feces and bile were screened and compared with the profiles of the blank urine, feces and bile and samples collected before administration. As shown in Figure 7, totally 15 metabolites (M1–M15) of ASD (M0) were observed in the urine, feces and bile after dosing. Notably, abundant metabolites derived from de-glycosylation (de-) methylation (de-) hydroxylation, de-carbonylation, de-carboxylation, hydroxymethylene loss and hydration were also observed. Characterization of these metabolites was described as below. Fourteen metabolites were screened in the feces, including M1: m/z795.5 (loss of C_5_H_8_O_4_ from M0), M2: m/z 765.5 (loss of C_6_H_10_O_5_ from M0), M3: m/z 633.5 (loss of C_6_H_10_O_5_ from M1), M4: m/z 603.5 (loss of C_6_H_10_O_5_ from M2), M5: m/z 471.5 (loss of C_17_H_28_O_14_ from M0), M6: m/z 943.5 (hydroxylation, M0+O), M7: m/z 897.5 (loss of hydroxymethylene from M0), M8: m/z 913.5 (loss of methylene from M0), M9: m/z 957.5 (hydroxylation and methylation, M0+CH_2_O), M10: m/z945.5 (hydrolysis, M0+H_2_O), M11: m/z 971.5 (hydroxylation and ethylation, M0+C_2_H_5_O), M12: m/z 899.5 (decarbonylation, M0-CO), M13: m/z 911.5 (dehydroxylation, M0-O), M14: m/z 941.5 (methylation, M0+CH_2_). Ten metabolites were screened in the urine, including M1, M2, M3, M4, M5, M9, M10, M11, M13, and M14. Ten metabolites were screened in the bile, including M1, M2, M3, M4, M5, M9, M11, M14, and M15 (decarboxylation, M0-CO_2_).

## Conclusion

In the present study, we reported a robust LC–MS/MS assay that is sensitive, selective and able to show good linearity of response and high precision for fast and accurate determination of ASD and its metabolites in biological specimens including the plasma, feces, urine and bile. The methods were successfully applied to the investigation of the bioavailability, metabolism, and excretion of ASD in rats. Meanwhile, the UPLC–HR/MS system was used to screen all possible metabolites in the urine, feces and bile, and compared them with blank samples collected before administration.

In summary, the pharmacokinetics, bioavailability, metabolism and excretion of ASD in rats were systematically evaluated for the first time in this study. According to the results of this study, the ultra-low oral bioavailability (0.025%) of ASD might be due to the poor gastrointestinal permeability, extensive pre-absorption degradation and biotransformation. The relative bioavailability could be increased to different extents by a series of interventions such as improvement of lipid solubility and intestinal permeability as well as inhibition of intestinal flora, but was not affected by P-gp inhibition. After iv administration, the urine and bile turned out to be the main excretion pathways of ASD. Fifteen possible metabolites were observed in the urine, feces and bile by UPLC-HR/MS analysis, and the metabolic pathway of ASD was explored to find out more about the metabolism.

## Data Availability

The original contributions presented in the study are included in the article/Supplementary Material, further inquiries can be directed to the corresponding authors.
